# 2722. Risk of Infectious Adverse Events of Venetoclax Therapy in the Treatment of Hematologic Malignancies: A Systematic Review and Bayesian Meta-Analysis of Randomized Controlled Trials

**DOI:** 10.1093/ofid/ofad500.2333

**Published:** 2023-11-27

**Authors:** Connor Prosty, Khaled Katergi, Alex Nguyen, Owen Luo, Mark Sorin, Vladimir Cherniak, Michael Sebag, Koray Demir, Emily McDonald, Todd C Lee, Matthew Cheng

**Affiliations:** McGill University, Montréal, Quebec, Canada; Université de Montreal, Montreal, Quebec, Canada; McGill University, Montréal, Quebec, Canada; McGill University, Montréal, Quebec, Canada; McGill University, Montréal, Quebec, Canada; McGill University Health Centre, Montreal, Quebec, Canada; McGill University Health Centre, Montreal, Quebec, Canada; Université de Montreal, Montreal, Quebec, Canada; McGill University Health Centre, Montreal, Quebec, Canada; McGill University, Montréal, Quebec, Canada; McGill University Health Centre, Montreal, Quebec, Canada

## Abstract

**Background:**

Venetoclax is a small molecule inhibitor of BCL-2 used in the treatment of acute myelogenous leukemia and chronic lymphocytic leukemia. Recent post-marketing studies on venetoclax suggested that it may predispose to opportunistic infections (OIs). We sought to systematically review the randomized controlled trial (RCT) evidence on venetoclax to assess whether it predisposes patients to infectious adverse events (IAEs) and neutropenia.

**Methods:**

We systematically reviewed the RCTs comparing venetoclax therapy to active (*i.e.,* a regimen with additional anti-cancer agents) or placebo controls in patients with hematologic malignancies, irrespective of treatment line. Data on IAEs and neutropenia were pooled by Bayesian meta-analysis and we computed the probability of any increased risk in infectious or neutropenic complications (Risk Ratio [RR] >1). Subgroup analyses were conducted based on treatment line, use of an inactive comparator, malignancy type, and prophylaxis strategy. Additionally, we pooled the incidence rates of fatal OIs between venetoclax and comparator arms and compared them by univariate meta-regression.

**Results:**

Seven RCTs were included comprising 2067 patients. In venetoclax versus comparator regimens there was a 81.1% probability of any increase in IAEs (RR=1.14, 95% Confidence Interval [95%CI]=0.76-1.66). The probabilities of venetoclax increasing the risk of grade 3-5 and fatal IAEs were 86.6% (RR=1.16, 95%CI=0.86-1.55) and 81.1% (RR=1.40, 95%CI=0.66-2.94), respectively. There was a 97.2% likelihood that venetoclax increased the risk of grade 3-5 neutropenia (RR=1.44, 95%CI=1.01-2.10). Venetoclax treatment did not increase the incidence of fatal OIs (P=0.55).

**Conclusion:**

Our results suggest that venetoclax likely increases the risk of high-grade neutropenia and IAEs overall, but not fatal OIs. However, our analyses did not identify any specific IAEs that would benefit from routine anti-infective prophylaxis or pre-emptive testing.

**Disclosures:**

**Michael Sebag, MD, PhD**, Amgen: Advisor/Consultant|BMS: Advisor/Consultant|Focus Therapeutics: Advisor/Consultant|Gilead: Advisor/Consultant|Janssen: Advisor/Consultant|Novartis: Advisor/Consultant|Pfizer: Advisor/Consultant|Sanofi: Advisor/Consultant **Matthew Cheng, MD**, Amplyx Pharmaceuticals: Grant/Research Support|AstraZeneca: Advisor/Consultant|AstraZeneca: Honoraria|Cidara Therapeutics: Grant/Research Support|GEn1E lifesciences: Advisor/Consultant|GEn1E lifesciences: Stocks/Bonds|Kanvas Biosciences, Inc.: Board Member|Kanvas Biosciences, Inc.: Pending patents|Kanvas Biosciences, Inc.: Ownership Interest|Merck: Honoraria|nomic bio: Advisor/Consultant|nomic bio: Stocks/Bonds|Pfizer: Honoraria|Scynexis Inc.: Grant/Research Support|Takeda: Advisor/Consultant|Takeda: Honoraria

